# A Novel System for Fabricating Microspheres with Microelectromechanical System-Based Bioprinting Technology

**DOI:** 10.34133/bmef.0076

**Published:** 2024-11-20

**Authors:** Yifeng Xu, Bao Jiang, Fangfang Liu, Hua Zhang, Dan Li, Xiaohui Tang, Xiuming Yang, Yan Sheng, Xuanye Wu, Nan Shi

**Affiliations:** ^1^ School of Microelectronics, Shanghai University, Shanghai 200000, China.; ^2^ Shanghai Industrial μ Technology Research Institute, Shanghai 200000, China.; ^3^ Suzhou Silicon jet Microelectronics Co. Ltd., Suzhou, Jiangsu Province 215000, China.; ^4^ Ruijin Hospital Shanghai Jiaotong University School of Medicine, Shanghai 200000, China.; ^5^ Institute of Translational Medicine, Shanghai University, Shanghai 200000, China.

## Abstract

**Objective and Impact Statement:** The microspheres were widely utilized in the field of life sciences, and we have developed an innovative microelectromechanical system (MEMS)-based bioprinting technology (MBT) system for the preparation of the microspheres. The microspheres can be automatically and high-throughput produced with this cutting-edge system. **Introduction and Methods:** This paper mainly introduced a novel, efficient, and cost-effective approach for the microsphere fabrication with the MBT system. In this work, the whole microsphere production equipment was built and the optimal conditions (like concentration, drying temperature, frequency, and voltage) for generating uniform hydroxypropyl cellulose–cyclosporine A (HPC-CsA) and poly-l-lactic acid (PLLA) microspheres were explored. **Results:** Results demonstrated that the optimal uniformity of HPC-CsA microspheres was achieved at 2% (w/v) HPC-CsA mixture, 45 °C (drying temperature), 1,000 Hz (frequency), and 25 V (voltage amplitude). CsA microspheres [coefficient of variation (CV): ~9%] are successfully synthesized, and the drug encapsulation rate was 84.8%. The methodology was further used to produce PLLA microspheres with a diameter of ~2.55 μm, and the best CV value achieved 6.84%. **Conclusion:** This investigation fully highlighted the integration of MEMS and bioprinting as a promising tool for the microsphere fabrication, and this MBT system had huge potential applications in pharmaceutical formulations and medical aesthetics.

## Introduction

Microspheres, characterized by their micrometer-level particle size, typically fall within the range of 1 to 250 μm. The diameters of those microspheres measured less than 500 nm are reviewed as nano-microspheres [1]. The microspheres can be divided into 3 types based on materials: polymer microspheres, natural polymer microspheres, and inorganic microspheres. As we know, polymer microspheres are widely utilized in the life sciences. Among the polymer microspheres, drug sustained-release microspheres and cosmetic medicine microspheres attract much attention, especially the materials and preparation methods. In recent decades, the degradable, biocompatible, and stable polymers are viewed as the optimal excipients of the life-science microspheres. The stabilization of the polymers ensures the slow release of the medicine in the microspheres, and the polymer microspheres are believed to be safe to patients because of the properties of biodegradation and biocompatibility [2,3]. Currently, the drug sustained-release and cosmetic medicine microspheres are normally produced through microencapsulation, such as emulsion process, microfluidics, and atomizing drying. In this project, we mainly developed a more effective and intelligent approach for the fabrication of drug and cosmetic microspheres compared to traditional methods.

As we know, the medicine dissolution rate is proportional to its surface area [4]. Microspheres inherently possess large specific surface area, which notably expedites the medicine dissolution rate. Microsphere-encapsulated medicine also has another benefit of achieving on-demand release that effectively addresses the following problems: (a) the abbreviated circulation duration of pharmaceuticals in the physiological milieu, (b) frequent medicine administrations, and (c) enhancing the efficiency of medicines [5,6]. Kim et al. [7] elucidated that the 24-h cumulative medicine release from nonporous microspheres was 6.2 ± 1.1%, whereas the cumulative release of porous microspheres was 85.6 ± 1.0%, with discernible pre-surge release in 24 h. The microsphere formation of medicine also improves its tolerance to light, heat, and acid-base environment and lengthens the shelf life of pharmaceuticals. Furthermore, microsphere-encapsulated medicines are helpful to enhance the treatment effect and cut down expenses. Compared to traditional drugs, the microsphere medicines have the advantage of transporting medicine to the target area and then releasing that achieved precise treatment, reducing the required drug dosage, and mitigating toxicity to healthy tissues and cells.

In recent decades, hyaluronic acid, crotoxin, and collagen are conventional materials in aesthetic medicine. Those materials are straightly injected into the skin of face for correction of wrinkles, filling of scar depression, and facial remodeling. However, collagen injection has a high risk of allergic reactions and hyaluronic acid requires the addition of a cross-linking agent, which probably leads to not entirely being degraded and absorbed. Regenerative medicine [such as poly-l-lactic acid (PLLA), polycaprolactone (PCL), and poly(lactic-co-glycolic acid) (PLGA) microspheres] attracts much attention in the medical beauty field because of its stimulation of collagen secretion, nontoxicity, nonirritating nature, and eventual metabolism into carbon dioxide and water in the human body. When regenerative medicine enters into the deep layers of the skin, it sustains inducing human fibroblasts to secrete and synthesize collagen for more than 2 years.

As we mentioned above, microsphere-based medicine has greater potentials than the traditional dosage form of medicine in some disease fields. However, the universal use of the microspheres in medical drugs is constrained by some elements: low uniformity, high preparation cost, industrialization amplification, inconsistent batch repeatability, and so on [8–11]. Therefore, it becomes crucial to develop a powerful tool for microsphere generation.

Traditional methods for preparing microspheres include phase separation, emulsification volatilization [12], and inkjet drying [13] microfluidics. Using the phase separation method to generate microspheres, it is difficult to avoid mutual collision and adhesion in the sphere-forming stage. In addition, the particle size of the microspheres is not highly uniform, and there is some organic solvent that remained in microspheres. The emulsification volatilization method is affected by many parameters, such as the solubility of the drug, the amount of polymer material, the selected organic solvent, the amount of emulsifier, the emulsification temperature, and the viscosity and ratio of the aqueous and oil phases. Therefore, the batch repeatability and process scale-up are the key issues in emulsification volatilization for microsphere generation. The spray drying method looks like an effective technique to obtain soluble fine powder [13]. Tomoda et al. [14] applied the spray drying method to fabricate adhesion microspheres for the treatment of mucositis by mixing carrageenan and allopurinol. Nevertheless, the spray drying method for microsphere preparation exhibits a relatively broad particle size distribution, and the drying temperature substantially influences the activity of medicine encapsulated inside microspheres [15]. Microfluidics-based chips have obvious advantage on the uniformity of microsphere sizes. Zhang et al. [16] proposed to adjust the microsphere yield and droplet size via surface treatment in the microchannels, which increased the droplet yield by 12.2% and reduced the impact of flow ratio on the microsphere yield by 19.1%. This method not only addressed the issue of low yield but also shortened the experiment process and made it less susceptible to environmental interference. This technology mainly optimized the yield of the microsphere generated with microfluidics, but the automation and integration of the system remain to be improved. Chen et al. [17] introduced an effective method combining microfluidics and interfacial instability to produce monodisperse PLGA-poly(ethylene glycol) (PEG)/PLGA microspheres with precisely controlled sizes and surface morphologies for tailored drug release. This effective technique could generate microspheres with a high uniformity and significantly enhanced the control of drug release. However, this device only maintains a high uniformity [coefficient of variation (CV) < 10%] when preparing 5- to 100-μm particles.

The main advantages of the microelectromechanical system (MEMS)-based bioprinting technology (MBT) system contained effective, precision machining, intelligent control, easy to mass production, microliter reactants system, bio-compatibility, and cost-effectiveness. Hence, our team introduced an automated and high-throughput system to produce microspheres with a one-step process via MBT. Our team successfully fabricated cyclosporine A (CsA) and PLLA microspheres with the MBT system. The microspheres were mainly characterized in terms of particle diameter, CV, grain sphericity, and encapsulation rate. By exploring and adjusting the polymer/medicine concentration, dry temperature, and the frequency and voltage of micropumps, the optimal condition for microsphere generation was figured out in this work. The particle diameter ranged from 2 to 5 μm (CV < 10%), and the encapsulation of CsA achieved ~85% with one step in this MBT. What is more, the throughput of particle production could reach 12 ml/min theoretically with this fully automated machine.

## Materials and Methods

### Reagents and equipment

The polymers in 2 types of the microspheres were hydroxypropyl cellulose (HPC) and PLLA. Dioxane was used as the organic solvent to dissolve the medicine, and polymers CsA, PLLA, HPC, and dioxane were purchased from CANSPEC.

The equipment utilized in this work contained semiconductor sprayers, dropwatcher [Shanghai Industrial μ Technology Research Institute (SITRI)], drying chamber (SITRI), collection films (SITRI), scanning electron microscopes (SEMs) (Zeiss, X-Max^N^), magnetron sputtering devices (Quorum, Q150RES), viscometers (RheoSense, μVISC), optical microscope (Olympus, STM7-CB), and ultraviolet (UV)–visible spectroscope (Thermo Fisher, Nanodrop One).

### MBT system

**Fig. 1.**
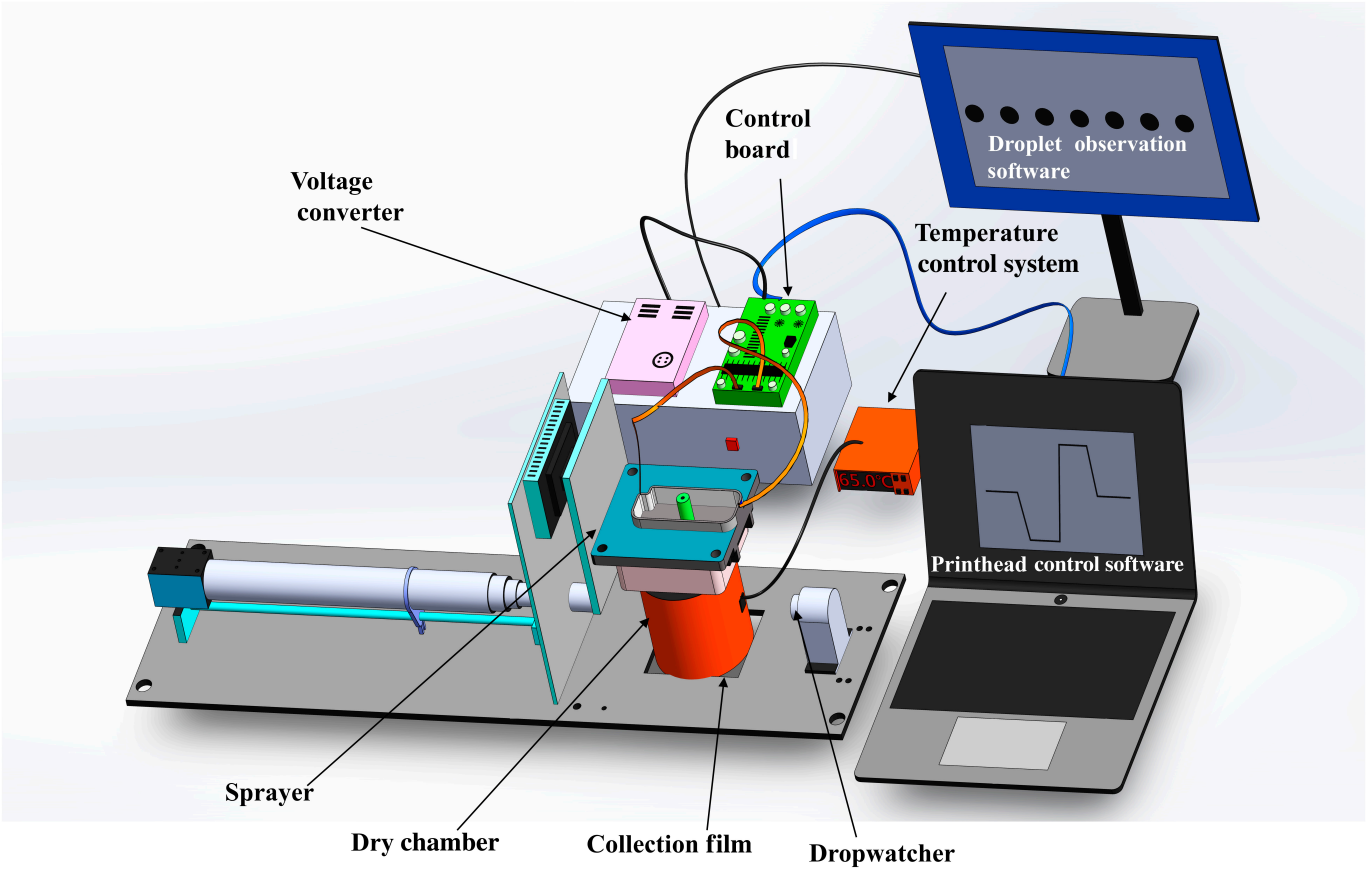
Schematic diagram of microsphere preparation machine (MBT).

Figure 1A depicts the whole schematic of the MBT system. Piezoelectric waveform was input into the control board and drove MEMS sprayers. Dropwatcher was utilized to gather and analyze the information (volume, velocity, deflection angle) of droplets generated from the active sprayers. The drying chamber was used to remove the solvent inside droplets and format particles, and the final microspheres were collected with hydrophobic films or bottles.

### Microsphere preparation

**Fig. 2.**
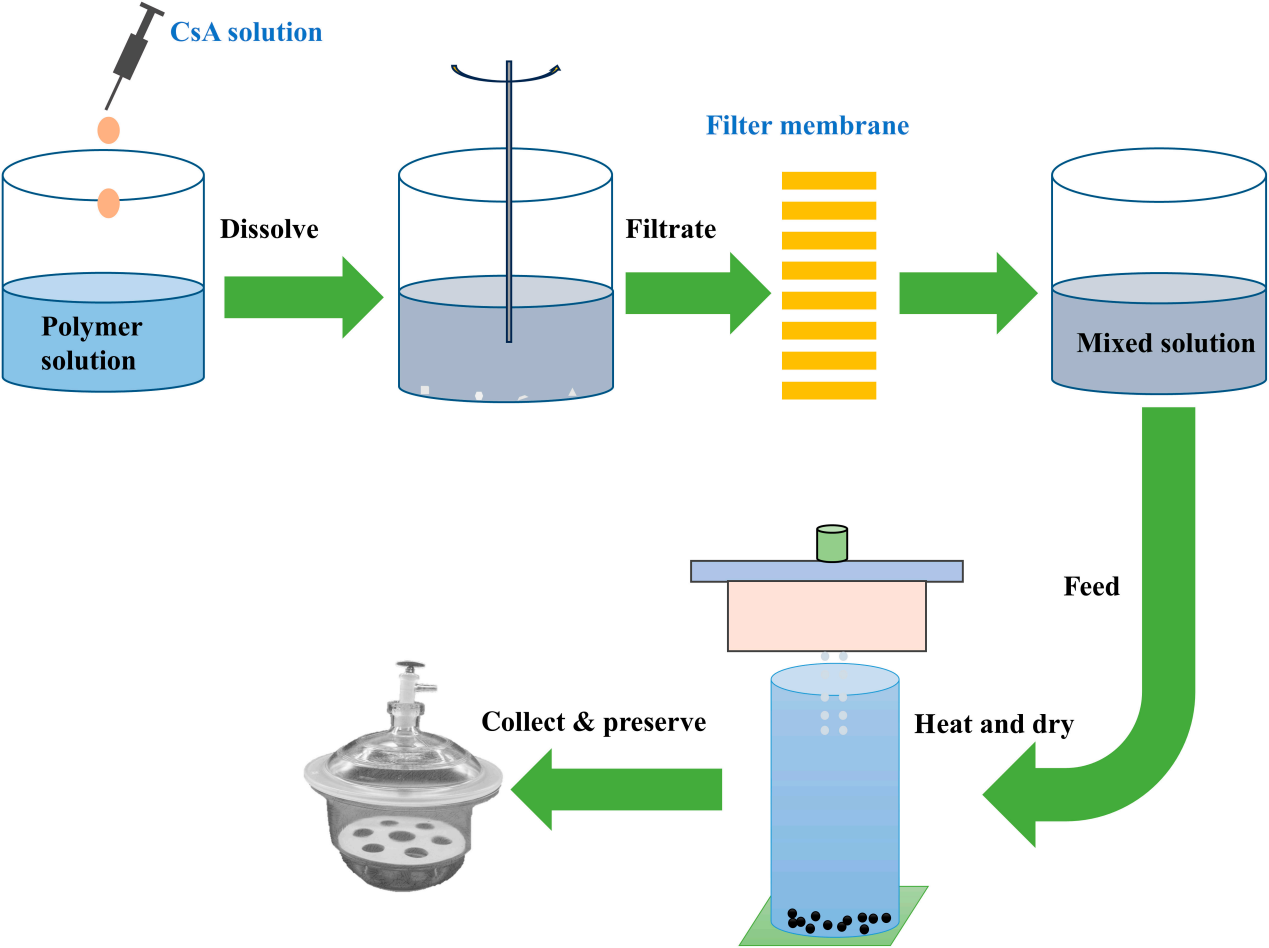
Schematic diagram of microsphere preparation process.

CsA (0.02 g) and HPC (0.38 g) were precisely weighed and dissolved in 2 and 18 ml of dioxane, respectively. Subsequently, the CsA and HPC-SSL (super super light) (a kind of low-viscosity hydroxypropylcellulose polymers) solution was mixed together. After stirring [1,000 rpm, room temperature (RT)] for 1 h, the mixed solution was filtered to remove the undissolved reactants. The filtered sample solution was collected and stored well for the next step [18]. Then, the sample solution was injected into the MEMS sprayer and the microspheres were produced after droplets flew through the drying chamber. The entire preparation process was fully illustrated in Fig. 2. Some properties of the microspheres were demonstrated via our self-developed image analysis methodology (“Particle analysis methodology” section). Furthermore, the 3-dimensional (3D) morphology of the microspheres was obtained with SEM.

### Key parameters for microsphere preparation

The concentration of sample was a pivotal parameter in particle preparation in this MBT system. Various concentrations would lead to different physicochemical property (viscosity, solubility, and surface tension), which should impact the formation of the microspheres. For example, the rise of viscosity would decrease the fluidity of the solution that helped maintain the spherical shape of droplets so that the viscosity should influence the morphology of the microspheres based on mass balance theory [19,20]. Theoretically, there was a positive correlation between the final diameter of the microspheres and the sample concentration [21].

Drying temperature was another crucial factor that significantly impacted the sphericity and size homogeneity of particles. Relatively low drying temperature would result in slow evaporation of solvent in particles and decrease the yield of the microspheres. However, excessively high drying temperatures were likely to cause rapid evaporation of the organic solvent that would result in the microspheres with less-rounded shapes, irregularities, or even cracking.

In this MBT device, piezoelectric-based micropumps were viewed as the tool to precisely transport fluid. The piezoelectric materials would deform once the voltage was applied. The piezoceramic plate deformed in response to an electric impulse to generate a pressure wave to eject the liquid. The output voltage applied to piezoelectric chip was precisely controlled by the waveform, so the waveform had important effects on the formation of droplets. These droplets were then dried in the chamber and gradually turned into microspheres. Therefore, the frequency and voltage amplitude of microchip should significantly affect the microsphere production. Compared to high frequency, relatively lower frequency lengthened the force duration, which helped format better spherical structure and reduce size discrepancies, but the yield and size of the particle would be lower and larger, respectively. On the other hand, particle collision and aggregation might happen on the condition of extra high frequency. The voltage amplitude would have an influence on the velocity, volume, deflection angle, and drying temperature of the microspheres. So, it was important to real-time observe and gather the droplet condition, which would help the researcher adjust and figure out the optimal waveform of output voltage and produce eligible microspheres. Dropwatcher with high-speed camera was viewed as an effective tool to collect the quantitative and qualitative information of droplets, like droplet geometric configuration, volume, velocity, and deflection angle.

In summary, this paper investigated the effects of substance concentration, drying temperature, frequency, and voltage amplitude of micropump on the generation of the microspheres in the MBT.

### Surface morphology

Before they were photographed with SEM (Zeiss, X-Max^N^), the microspheres were gold plated with a magnetron sputtering device (Quorum, Q150RES) for 20 min.

### Measurement of the viscosity of solution

The viscosity of sample solution was assessed with a viscometer (RheoSense, μVISC). Note that each sample should be tested thrice to minimize errors.

### Particle analysis methodology

Normally, a laser particle size analyzer was used to analyze the volume distribution of particles. In this work, cost-effective, accurate, and rapid Python-based image [the original microsphere image was taken under an optical microscope (Olympus, STM7-CB)] analysis was used as an alternative method for gathering microsphere size information. The Simple Blob Detector feature point detection was used in this method. The kernel density estimation map was generated with Kdeplot function based on microspheres’ radius to estimate the size distribution. The NumPy Library (NP) function was employed to calculate the CV values of microsphere radius. Pixel from 100 to 650, minimum roundness of 0.6, convexity filter of 0.65, inertia ratio filter of 0.3, and bandwidth of 0.13 were set based on our database.

### Encapsulation rate of pharmaceutical microspheres

Some CsA microspheres were weighted and dissolved in dioxane solution. Then, the sample was detected with 210-nm wavelength in the UV spectrophotometer (Thermo Fisher, Nanodrop One), and the amount of CsA in solution was equal to the amount of CsA encapsulated inside microspheres. Then, various concentrations of CsA solution were measured and the standard curve of UV absorbance amplitude and medicine concentration was built. Finally, the amount of CsA dissolved in solution could be calculated based on the standard curve and the actual mass of CsA encapsulated in microspheres was figured out. The encapsulation rate was figured out with the following encapsulation rate formula: encapsulation rate = (actual mass of medicine in microspheres/theoretical mass of medicine in microspheres) × 100%.

## Results and Discussion

### Temperature evaluation of drying chamber

**Fig. 3.**
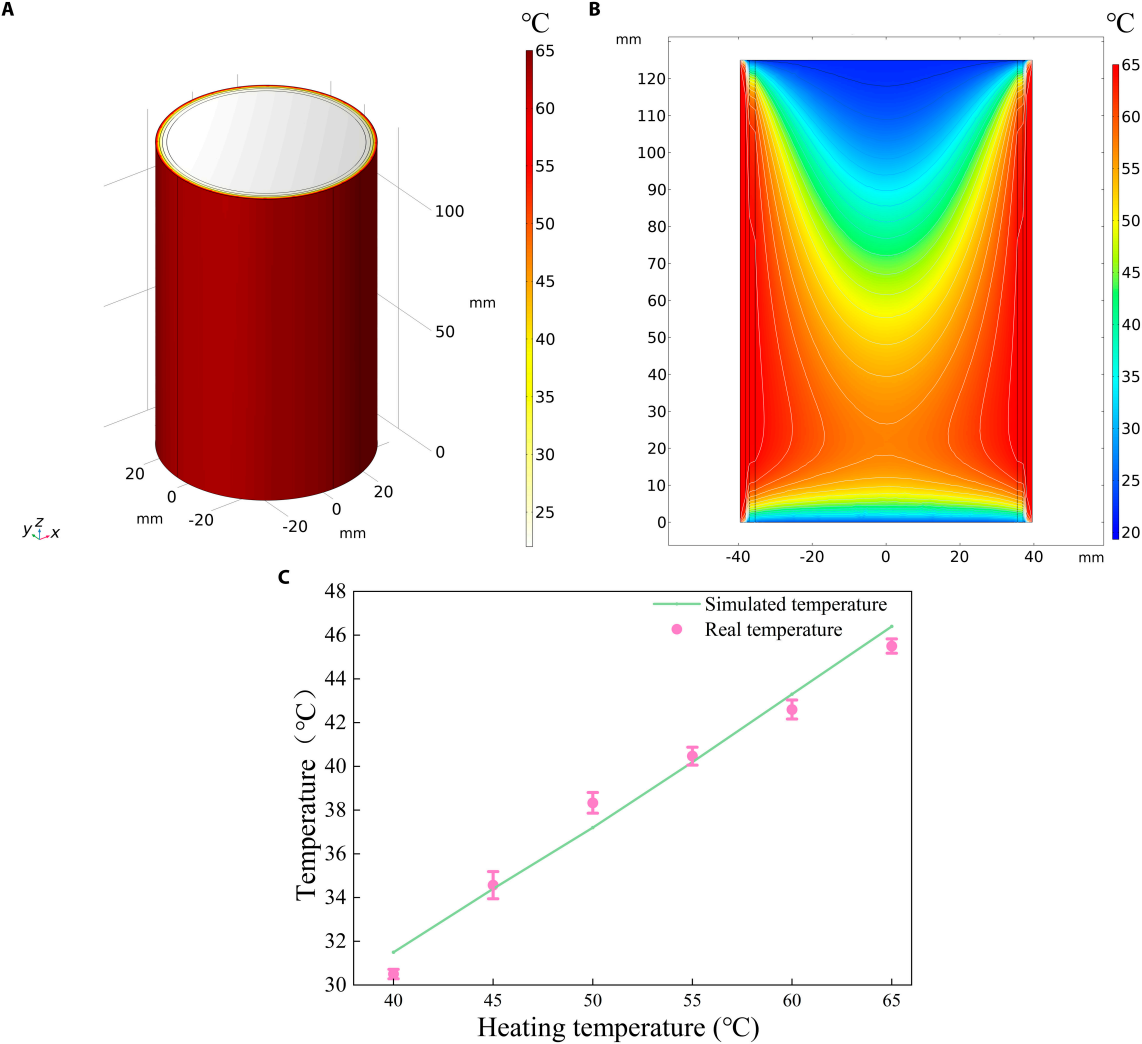
(A) Simulated temperature distribution. (B) Cross-sectional temperature distribution. (C) Comparison of actual and simulated temperatures.

The temperature of the drying chamber critically affects microsphere formation, such as the particle size distribution, sphericity, and drug encapsulation efficiency [21]. To demonstrate the distribution of temperature inside the drying chamber, actual measurement and simulation tool (COMSOL) were integrated to solve this problem. Figure 3A illustrates the geometry of the cylindrical drying chamber (72 mm in diameter, 125 mm in height), while Fig. 3B shows the temperature distribution inside the drying chamber across the sectional view. There was a difference of 20 °C between the center and the external area when the heating temperature was set at 65 °C. The actual temperature was well consistent with the simulated temperature (difference < 1 °C), as shown in Fig. 3C, which significantly indicated the reliability of this temperature simulation and evaluation system. This temperature evaluation system had important influence on exploring the effect of temperature on microsphere formation in this work.

### Effects of sample concentrations on microsphere morphology

**Fig. 4.**
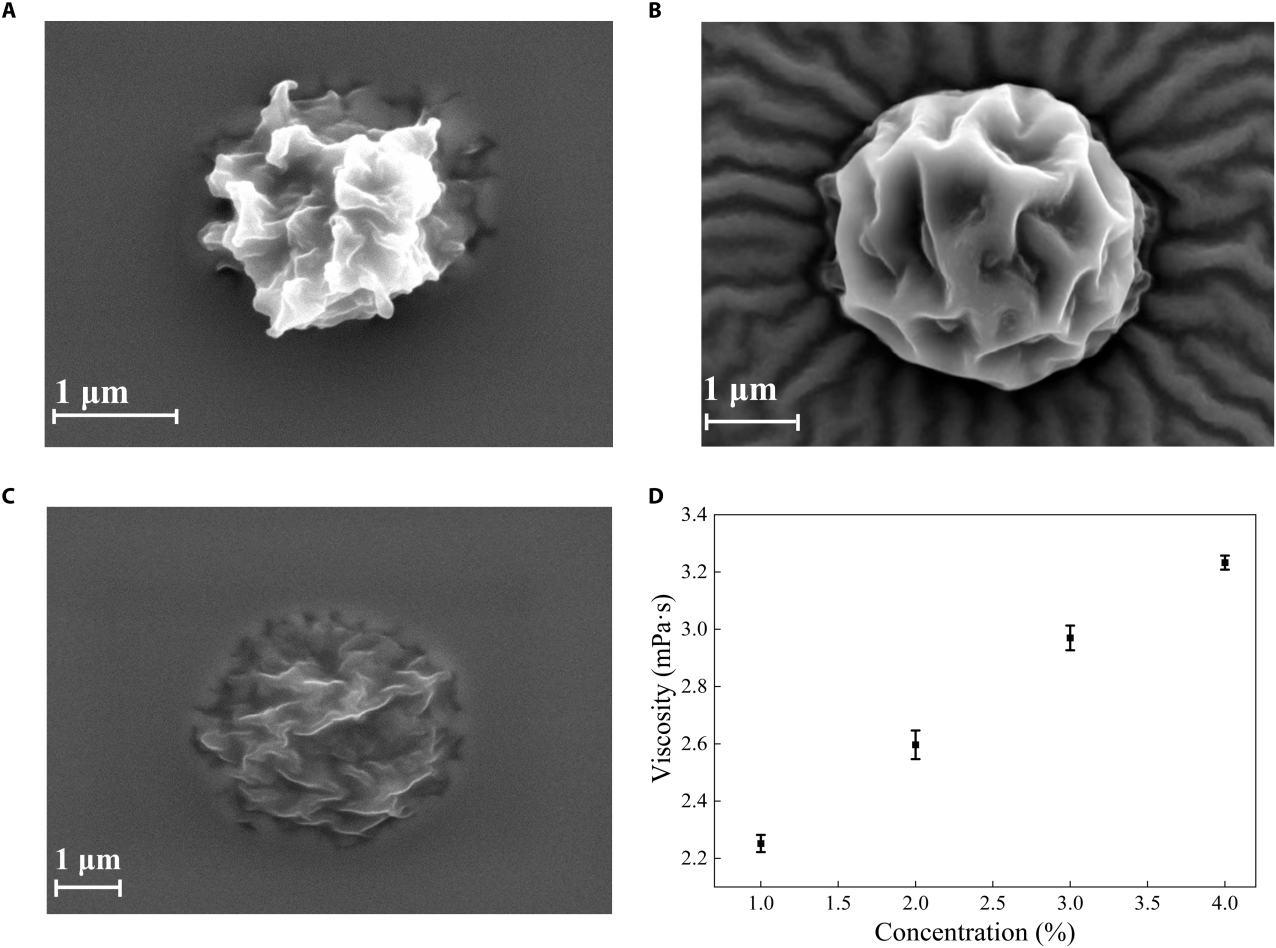
CsA microspheres at various concentrations: (A) 1%, (B) 2%, and (C) 3%. (D) Viscosity of CsA at different concentrations (RT).

Keeping other parameters (dry temperature, frequency, and voltage) constant, the effects of sample concentrations (w/v) on microsphere morphology were mainly explored here. Figure 4A shows that the microspheres lost the spherical morphology when the concentration of HPC-CsA was 1% and the solution viscosity was 2.25 mPa·s (Fig. 4D). It was estimated that the low amount of polymer could not support the structure. Figure 4C demonstrates that 3% (2.97 mPa·s) was not a suitable concentration for microsphere generation because the higher concentration of polymer significantly decreased the flowability of solution and improved surface tension. The microsphere manifested clearly discernible 3D spherical shape at the concentration of 2% (Fig. 4B).

### Effects of drying temperatures, micropump frequency, and voltage on microsphere fabrication

**Fig. 5.**
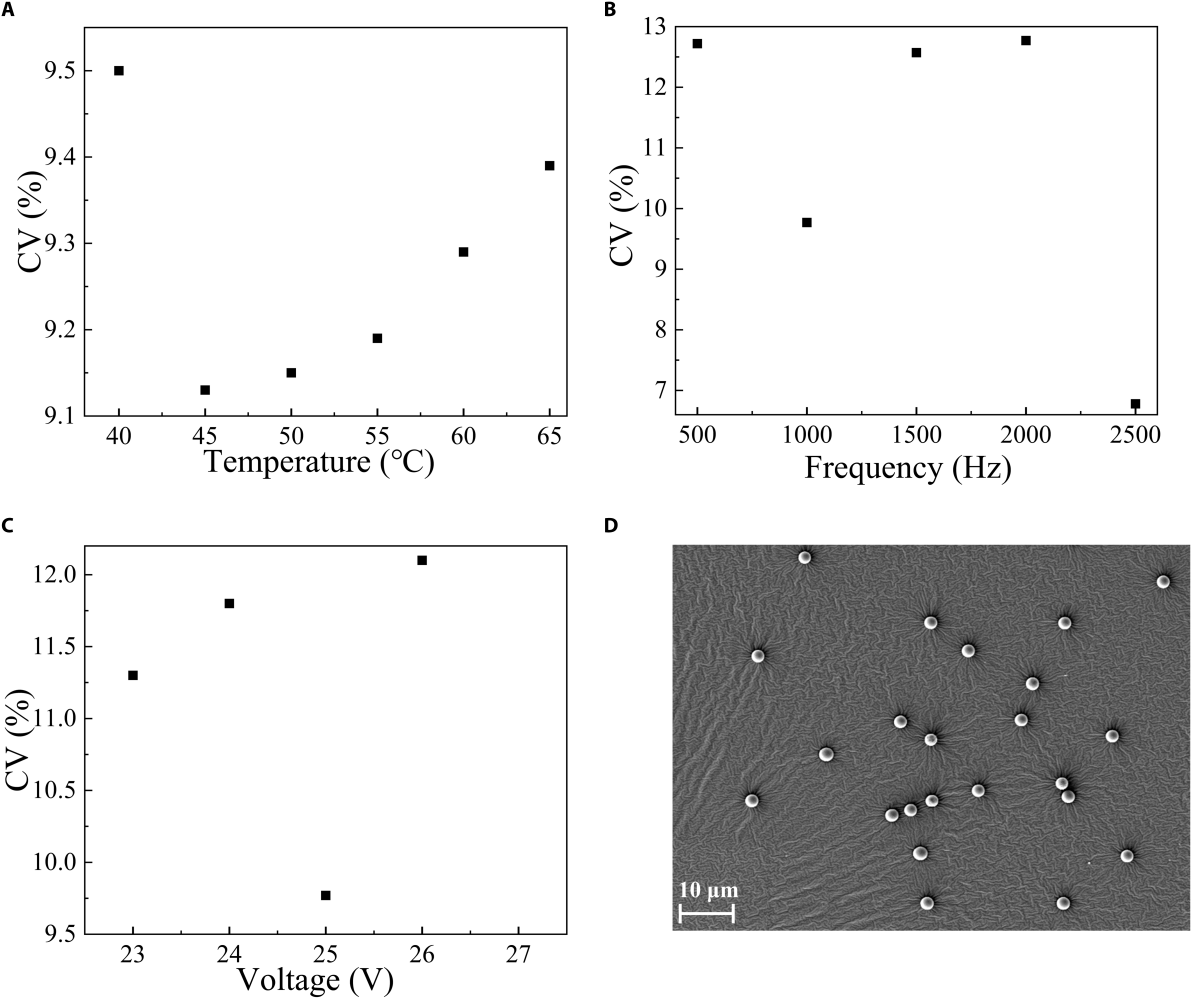
(A to C) CV values (size distribution) of CsA microspheres at different temperatures, frequencies, and voltages. (D) CsA microspheres at the optimal condition.

The CsA microspheres prepared at various temperatures (40, 45, 50, 55, 60, and 65 °C) were analyzed with the image-based method described in the “Particle analysis methodology” section. As shown in Fig. 5A, the lowest CV value of 9.13% was found at 45 °C, which represents the narrowest range of particle distribution. The CV value increased when the temperature rose from 45 to 65 °C. Interestingly, some pores were found on the surface of the microspheres at 65 °C and there were no pores in those microspheres at 45 °C.

The effects of frequencies of micropump on microspheres were explored in this work including 500, 1,000, 1,500, 2,000, and 2,500 Hz. In Fig. 5B, the best particle uniformity appeared at 2,500 Hz and the CV value was 6.78%. However, due to the relatively high frequency, the formed microspheres contained a large amount of organic solvent (the droplets cannot dry completely to form microspheres). The CV associated with 1,000 Hz is the next best, at 9.77%. Therefore, subsequent research will focus on exploring other parameters affecting the uniformity of CsA microspheres at a piezoelectric frequency of 1,000 Hz.

Based on our preliminary experiment, small satellite droplets would generate when the micropump voltage amplitude was lower than 22 V or above 26 V. Therefore, this work focused on digging the influence of voltage on microsphere formation at 23, 24, 25, and 26 V. As shown in Fig. 5C, the best uniformity of CsA microspheres was achieved with a CV of 9.77% at 25 V. Figure 5D shows the microsphere produced at the optimal condition (2% w/t, 45 °C, 1,000 Hz, 25 V).

### Durg encapsulation rate of CsA microspheres

**Fig. 6.**
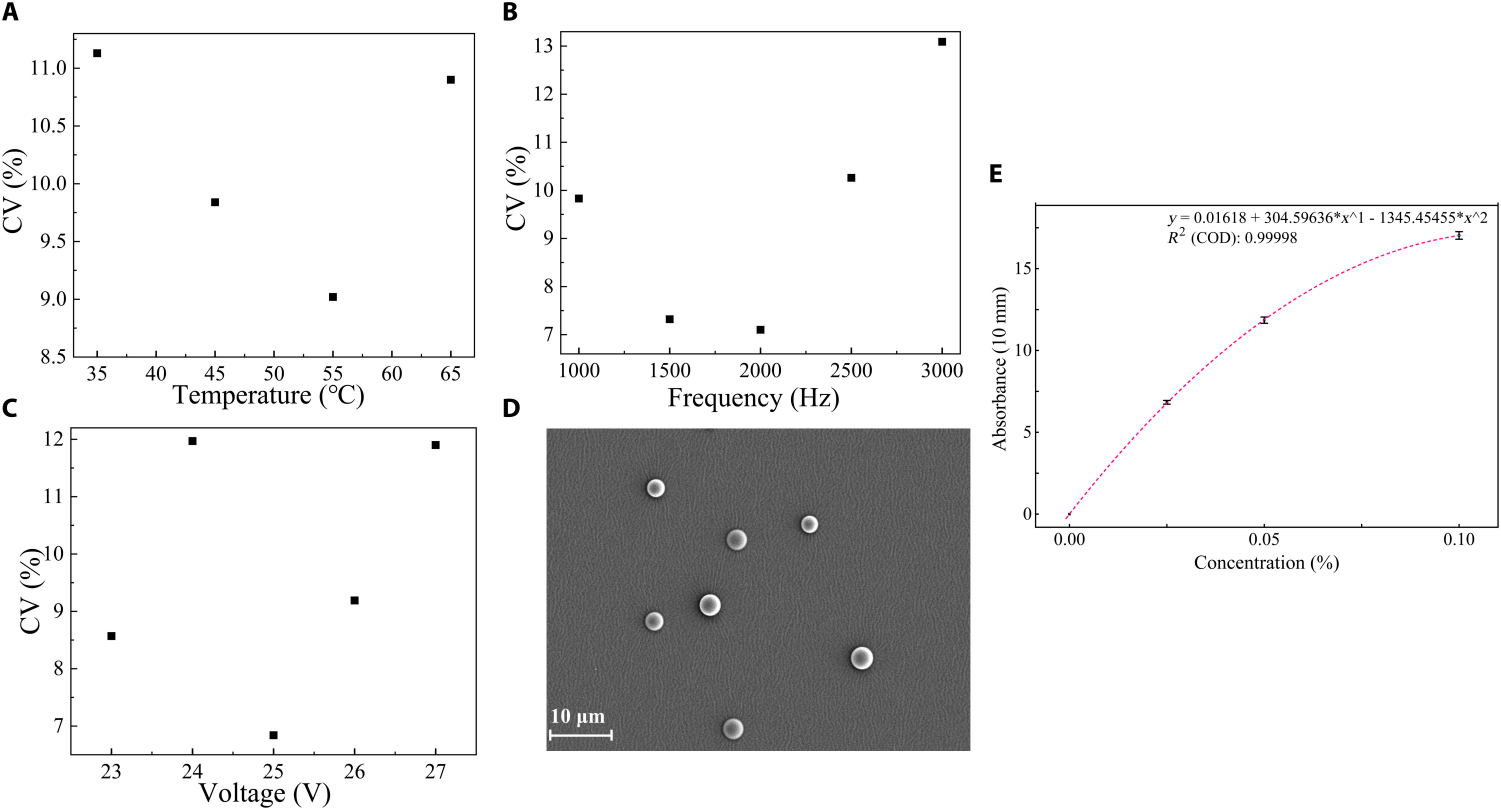
(A to C) CV values (size distribution) of PLLA microspheres at different temperatures, frequencies, and voltages. (D) Microspheres prepared with optimal parameters (0.1% w/v, 55 °C, 2,000 Hz, and 25 V). (E) Standard curve of UV absorbance versus CsA concentration.

The drug encapsulation rate of the pharmaceutical microspheres was measured following the “Encapsulation rate of pharmaceutical microspheres” section. As shown in Fig. 6A, the standard curve well fit with polynomial simulation (quadratic) and *R*^2^ was equal to 0.99998. The final concentration of CsA in sample solution was 0.0106% (w/v) according to the standard curve, and the encapsulation rate of CsA in microspheres was 84.8% after calculation and transformation.

### PLLA microsphere

To demonstrate the multifunction and scalability of the MBT system, PLLA microspheres were selected to be fabricated with MBT for cosmetic applications. The PLLA microspheres were formatted, collected, and analyzed at various temperatures (35, 45, 55, and 65 °C) with the method described in the “Particle analysis methodology” section. As shown in Fig. 6A, the optimal uniformity of particle size (~2.5 μm in diameter) appeared at 55 °C (the central temperature of the drying chamber was 40.2 °C), with a CV of 9.02%. Similarly, the best suitable frequency and voltage of micropump for particle preparation were found to be 2,000 Hz and 25 V, with corresponding CV values of 7.10% and 6.84%, respectively (Fig. 6B and C). In addition, the PLLA microspheres produced at the optimal condition (0.1% w/t, 55 °C, 2,000 Hz, 25 V) in this work were depicted in Fig. 6D. These compelling results significantly validate the capability of the MBT system to consistently and effectively fabricate high uniform microspheres, which demonstrates its scalability and reliability across a diverse range of applications in the life sciences.

## Conclusion

This paper presented a novel, high-uniformity (CV < 10%), high-throughput, low-cost, one-step microsphere preparation technique (MBT). Both CsA and PLLA microspheres were successfully produced with the MBT system in this work. The influences of material concentration, drying temperature, frequency, and voltage of micropump on the morphology and size distribution of CsA and PLLA microspheres were investigated here. We found that the optimal condition for CsA microspheres (~3 μm) was 2% (w/v), 45 °C, 1,000 Hz, and 25 V. For PLLA microspheres (~2.5 μm), the optimal uniformity was achieved at 0.1%, 55 °C, 2,000 Hz, and 25 V. What is more, the drug encapsulation rate of CsA microspheres was 84.8%. This paper demonstrated a promising and cutting-edge technique for microsphere preparation and solved some difficult problems in traditional methods, which would significantly help widen the applications of the polymer microspheres.

## Data Availability

Data will be made available on request.
